# Exploring ethnic differences in understanding of self-rated health among persons of Turkish, Bosnian and German origin

**DOI:** 10.1186/s13104-018-4019-9

**Published:** 2018-12-18

**Authors:** Kalpani Wijekoon Wijekoon Mudiyanselage, Florence Samkange-Zeeb, Tilman Brand, Hajo Zeeb

**Affiliations:** 1 Faculty 11, Human and Health Sciences, University of Bremen, Grazer Str.2, 28359 Bremen, Germany; 2 Department of Prevention and Evaluation, Leibniz Institute for Prevention Research and Epidemiology – BIPS, Achterstr. 30, 28359 Bremen, Germany

**Keywords:** Self-rated health status, Ethnicity, Qualitative content analysis, Turkish, Bosnian, German, Cognitive interviewing

## Abstract

**Objective:**

Self-rated health (SRH) is a widely used indictor of the subjective health status in population-based studies. However, differences in the reporting style across ethnic groups may limit the predictive ability of SRH for objective health outcomes. As part of the preparation phase of the UPWEB (understanding the practice and developing the concept of welfare bricolage) project, this study explored ethnic differences in the understanding of self-rated health among persons of Turkish, Bosnian and German origin, living in two northern Germany cities, Bremen and Hamburg.

**Results:**

Thirty persons, 10 per ethnic group, aged 32–82 years, took part in the assessment based on cognitive interviewing. All three ethnic groups defined SRH as the absence or presence of visible or non-visible disturbances and/or deviations from the norm, the ability or limited ability to act as well as the result of specific behaviours. However, only participants from the two migrant groups referred to community cohesion and religious or traditional beliefs as aspects of their SRH, indicating a systematic difference in the understanding of this question.

**Electronic supplementary material:**

The online version of this article (10.1186/s13104-018-4019-9) contains supplementary material, which is available to authorized users.

## Introduction

Self-rated health (SRH) is a widely used measure in epidemiologic research, which is practical, cost-efficient and serves as a means to assess the subjective health status and predict the objective health status of a population [1, 2]. While some research has suggested that migrant populations report an overall worse subjective health compared to their native counterparts [3], there is also evidence showing lower mortality rates for many migrant groups [4–6]—a contradiction that is termed *the immigrant paradox* [7]. Differences in the reporting style across cultural groups could partly explain this paradox [8–11]. For example, in a study comparing first-generation residents of Turkish and Moroccan origin and native Dutch communities in the Netherlands, diabetes was associated with a poor SRH among Turks, but not among the Dutch. Furthermore, general hospital admissions were associated with fair SRH by the Dutch, but not by the Moroccans [4].

This qualitative study was developed as part of the preparation phase for the UPWEB study, which was conducted in 4 European cities, including Bremen. Our aim was hence to explore ethnic differences in the understanding of the SRH-questions. To enable the comparison of possible differences between a large and a small migrant group, and not only between migrants and non-migrants, we recruited German as well as first-generation Bosnian and Turkish participants (n = 10 for each ethnic group). Turkish migrants represent the largest single group of foreign-born persons in Germany (12%), while Bosnians represent only 2% of the foreign-born population [12].

## Main text

### Methods

#### Study population

A total of 30 participants were recruited in two German cities: Hamburg and Bremen, using a convenience sampling method. The number of participants is based on recommendations by Dworkin, indicating that a minimum of 25 to 30 participants would approximately enable a saturation of data in a qualitative study [13]. To ensure that participants will have had some contact with the German health-care system, each migrant participant was required to have lived in Germany for at least 5 years. Further, an even distribution of gender was aimed for in each group, as gender has been found to influence SRH-outcomes [14].

#### Data collection

During face-to-face interviews held in the homes of the participants, quantitative as well as qualitative data were collected. The interviews were held in the German language.

Quantitative data included socio-demographic information as well as assessments of reported SRH, severity of somatic symptoms, health literacy and self-reported German language competences. Participants with low language competences were only included if they could express their understanding of the SRH question during the interview. Level of education was classified into three groups according to the International Standard Classification of Education-1997 (low 0–2; medium 3–4; high 5–6) [15]. SRH was assessed based on the first question of the 36-item short form health survey, with participants rating their general health on a five-point scale from excellent to poor [16]. Participants were also asked if they suffered from any chronic conditions and the associated functional limitations. The severity of somatic symptoms was evaluated using the Patient Health Questionnaire-15 [17] and each item was scored on a 3-point scale from 0 = *not bothered at all* to 2 = *bothered a lot.* Health literacy was assessed using the six-item short version of the European Health Literacy Survey Questionnaire [18]. Participants rated how easy it is for them to find, understand, evaluate and use health information on a 4-point scale from 1 = *very difficult* to 4 = *very easy*.

Qualitative data were collected following an interview guide that was developed in accordance with the methods of cognitive interviewing [19].

The *target question* used in this study is the afore-mentioned SRH question. To understand mental processes of the responder when answering the question, the following *verbal probing question* was used: *“What were you thinking when answering the question?”* [19].

Additionally, *thinking*-*aloud probing questions* were used to gather mental processes of the responder when reading a question: *“What needs to happen, that you have… (one option up/one option down) health status”* [19].

All interviews were transcribed according to Hoffmann-Riem’s transcription rules [20].

#### Analysis

For each ethnic group, absolute frequencies, means, standard deviations and scores of the standardized measurements were calculated for the respective variables using Microsoft Excel.

For the assessment of somatic symptoms, the scores for each item were summed up for each subject and could range from 0 to 30 in total. The level of severity was then categorised as follows: minimal 0–4 points, low 5–9, medium 10–14, and high 15–30 [21].

For health literacy, the mean of the scores for each item was calculated for each individual. The new variable was then recoded and the level of health literacy categorised as follows: 1.00–2.00 = 0 (low), 2.01–3.00 = 1 (medium), 3.01–4.00 = 2 (high) [22].

Qualitative data were analysed according to Mayring’s content analysis approach [23].

As the interviews were conducted in German, the selected transcript sections where participants responded to the respective questions were translated into English.

Each translated paragraph was then paraphrased. Throughout paraphrasing, all unnecessary or repetitive text passages were deleted to focus on the substantial information. Thereafter, subordinate categories were formed by directly generalizing the paraphrased sentences. Paraphrases similar in nature were allocated to the same subordinate categories. For example, descriptions that included any form of symptomatic sensations such as pain were generalized throughout the subordinate category *presence of subjective symptoms.*

Subordinate categories that related to the same assertion were again grouped together by formulating superordinate categories. For instance, all subordinate categories that included descriptions of symptoms, diseases and norm-deviations were put into the superordinate category *visible or non*-*visible disturbance and/or deviation from the norm* (Additional file 1). The raw material was abstracted throughout the development of subordinate and superordinate categories as this is said to be an ideal way of offering a transparent corpus while at the same time preserving the quality of the raw material [23]. The identification of subordinate categories was performed by one researcher (KWWM) and then reviewed and discussed with a second researcher (TB).

The SRH response options were grouped into two partly overlapping subsets of higher (excellent, very good, good) and lower SRH (good, fair, poor), in order to analyse contrasts and similarities in SRH-definitions across ethnic groups and levels of SRH (Additional file 2).

### Results

More than half of the participants were female, and the mean age was 52.7 years. In comparison to the migrant populations, Germans were generally older, had higher educational and professional qualifications and better health literacy competences (Table 1).

**Table 1:** Socio-demographic and health-related characteristics of the 30 study participants

	German (N = 10)	Turkish (N = 10)	Bosnian (N = 10)	Total study population (N = 30)
Sex				
Female	7	5	4	16
Male	3	5	6	14
Age (mean)	64.5 (SD = 16.16)	42.7 (SD = 4.6)	50.8 (SD = 11.5)	Mean = 52.6 Range = 32–82 SD = 14.47
Self-reported health				
Excellent/very good	3	3	6	
Good	4	4	3	
Fair/poor	3	3	1	
Presence of chronic conditions	6	6	1	
Functional limitations				
Severely limited	1	2	0	
Limited, but not severely	5	2	0	
Not limited at all	0	2	1	
Somatic symptom severity				
Minimal	2	0	6	
Low	4	4	2	
Medium	3	4	1	
High	1	2	1	
Mean severity	Score: 2.3 Minimal (SD = 0.95)	Score:2.8 Minimal (SD = 0.79)	Score: 1.7 Minimal (SD = 1.03)	
Educational level				
Low	0	2	2	
Medium	5	5	4	
High	5	3	4	
Self-reported language competence				
High		5	5	
Medium		4	4	
Low		1	1	
Health literacy				
High	7	5	6	
Medium	3	3	3	
Low	0	2	1	

Across all three ethnic groups, SRH was defined as the absence or presence of visible or non-visible disturbances and/or deviations from the norm, the ability or limited ability to act as well as the result of specific behaviours. Among others, differences between the German and the two migrant groups were observed regarding the role of the social community and that of religious or spiritual practices. Both Turkish and Bosnian participants included having a good relation to the social community in their definitions for higher SRH-status, defining lower SRH-status as being related to a disturbed well-being of the social community, including financial instability (Table 2) (Additional file 1).

**Table 2:** Differences and similarities in SRH-definitions among the German, Bosnian and Turkish participants

Overlapping aspects of SRH definitions across the three ethnic groups	Differences in SRH definitions between the German and migrant groups
Higher SRH-options	
Absence of visible or non-visible disturbances and/or deviations from the norm in terms of… Comparison Absence of disease Absence of subjective symptoms Presence of subjective well-being	Good relation to social community
Freedom in the ability to act in terms of… Performance level and role fulfilment Having no restrictions	
Result of specific behaviours in terms of … Healthy lifestyles Treatments Relaxation	Result of specific behaviours in terms of … Religious or spiritual practices
Lower SRH-options (good, fair, poor)	
Visible or non-visible disturbances and/or deviations from the norm in terms of… Treatable and/or tolerable symptoms or disease Presence of disease Presence of subjective symptoms	Result of specific behaviours in terms of… Unhealthy lifestyles Work
Limitation in the ability to act in terms of… Hindrance in performance level and role-fulfilment Dependency	Disturbed well-being of social community

Besides the absence or presence of certain disturbances in the form of symptoms or diseases, norm-comparisons, for instance in one’s surrounding, were set to justify higher SRH-options. For example, one participant reported *“I don’t have little ailments like others”* (Additional file 1). Higher SRH-options were generally associated with the result of specific behaviours such as treatments for a specific disease or symptoms, more relaxation, and a healthy life-style including physical activity and a healthy diet.

Regarding freedom or limitation in the ability to act, one migrant participant pointed out how work negatively impacted her health, leading to a lower SRH-status assessment: *“Because my back is paining […] because I am working, waking up half past four”* (Additional file 1). In addition, across all ethnic groups, participants referred to higher SRH as not being restricted and lower SRH to being dependent in everyday-life, for example due to the need of external help or medication.

In contrast to the German group, migrants included unhealthy lifestyles in the form of unhealthy diet, decreased physical activity and weight increase in their SRH-definitions. Furthermore, when asked to explain what needs to happen to reach a higher SRH status, migrants also mentioned specific behaviours in the form of religious acts such as praying or natural medication: “*I take other things, that for example* […] *old medicine”* (Additional file 1).

### Discussion

The results show that the three ethnic groups generally defined the SRH options similarly. Furthermore, our findings are consistent with prior research by Garbarski et al. showing that the understanding of SRH includes factors such as health conditions, health behaviour, physical state and functioning, comparative statements and descriptions of feelings. Interestingly, while family background, socioeconomic circumstances and spirituality did not play an important role in the study by Garbarski et al. [24] these were relevant aspects of SRH in our study.

In contrast to the Germany study group, both migrant groups included the category *good relation to social community* in their definitions for higher SRH-status and the categories *result of specific behaviours* and *disturbed well*-*being of social community* in the definitions for lower SRH-status.

Specific behaviours in form of acts of praying and intakes of natural medicine were associated with higher SRH-status assessments among the migrant groups. This is in line with observations made by Kizilhan and Bermejo [25], that spirituality and religion plays a role among some migrant groups when it comes to how one feels. Our findings are supported by the fact that religion plays a central part in the countries and communities of origin of both migrant groups in our study [26, 27].

Although only one migrant referred to the negative effects of work on health, other studies have observed that migrant populations are often subjected to precarious working conditions [3]. These can increase the risk of somatic and mental health problems [28], thereby contributing towards a negative assessment of SRH.

Furthermore, only migrant participants included the well-being and cohesion within the social community into their SRH definitions. This could be interpreted as a sign of a collective orientation, that is, placing personal needs secondary to the well-being of the community [29]. The fact that none of the German participants defined SRH-status based on collective values or religious aspects indicates the presence of a systematic difference across the three groups in the understanding of the SRH question.

The majority of studies in Germany comparing migrant populations to the native population focus on migrant populations large in numbers, such as those originating from Turkey or from the former Soviet Union [30–32]. There are hardly any health studies focussing on persons originating from Bosnia in Germany. Comparing the three ethnic groups not only enabled the comparison between migrants and non-migrants, but more importantly, between two migrant groups different in size.

### Conclusion

Our results indicate that while the SRH question is to a large extent similarly understood across ethnic groups, there are differences regarding the importance placed on social cohesion and community well-being. In contrast to the German study group, values of collectivism appear to play a more significant role in the SRH assessments of the two migrant groups.

Hence, SRH-status should be used with caution in multicultural populations as there appears to be a systematic way of understanding the SRH-options according to one’s ethnic background. Future research should try to quantify the extent to which collective orientations influence the rating of the SRH question.

## Limitations

The fact that the study population comprised a convenience sample limits the generalizability of our findings. Further, the German study population comprised mostly women and was generally older than the migrant groups. This could have influenced the way SRH was assessed, partly explaining the differences in the understanding of SRH-status between the ethnic groups [14, 33]. Although there were different levels of German language competency among the interviewees, this was not a major concern in the current study. Newly arrived migrants and those with very low German language skills might provide different insights, and further investigations should include even more diverse samples.

## Additional files

**Additional file 1.** Supporting quotes for each subordinate and the according superordinate definition categories given for higher and lower SRH-options.

**Additional file 2.** Allocation of subordinate definition categories among the higher (excellent, very good, good) and lower SRH options (good, fair, poor) for each ethnic group.

## Abbreviations

SRH: self-rated health
UPWEB: understanding the practice and developing the concept of welfare bricolage
