# Supplementary material for: Exploring ethnic differences in understanding of self-rated health among persons of Turkish, Bosnian and German origin
Source: BMC Res Notes. 2018 Dec 18;11:903. doi: 10.1186/s13104-018-4019-9 (PMC6299561; doi:10.1186/s13104-018-4019-9)
Supplement: Supplementary file 1 — Additional file 1. Supporting quotes for each subordinate and the according superordinate definition categories given for higher and lower SRH-options. [file 13104_2018_4019_MOESM1_ESM.pdf]

Additional File 1 – Supporting quotes for each subordinate and the according superordinate definition categories given for higher and lower SRH-options

A. Supporting Quotes for higher SRH-options (excellent, very good, good)

Tables 1A- 4A: Absence of visible or non-visible disturbances and/or norm equivalence

Table 1A Comparison

Superordinate category<br>Absence of visible or non-visible disturbances and/or norm equivalence				
Transcript	Original Quote	Translated Quote	Paraphrasing	Generalization
994, ll.21-22 German	„Und, dass ich nicht so wie andere Menschen Wehwehchen habe“.	“And, that I don’t have little ailments like others”.	Compared to other people who suffer from small pains, I am healthy.	Comparison with surrounding.
827, ll.23-24 German	„Interviewer: Was müsste passieren, in Ihrem Leben, damit Sie ein sehr gut ankreuzen können?“<br><br>„Befragter: das wird nicht mehr passieren, dann müsste ich jünger sein.“	“I: What needs to happen in your life, that you can tick very good?”<br><br>„Respondent: That won’t happen anymore, therefore I need to be younger “	Very good health status is not possible in her age-class.	Comparison with age-class

666, l.15 Bosnian	„Sehr gut. Für mein Alter.“	“Very good. For my age.”	I feel very good for my age.	Comparison with age-class.
333, ll.29-31 Bosnian	„aber ausgezeichnet, ich sag mal so, es ist in unserem Alter, beziehungsweise (…) man sagt sowas selten, man sagt entweder gut oder selten ausgezeichnet.“.	„ but excellent, let me put it like that, it is in our age, or rather (…) you say something like that only rarely, you say either good or rarely excellent.”	It is very rare to access excellent health in my age.	Comparison with age-class.

Table 2A Absence of disease

Superordinate category<br>Absence of visible or non-visible disturbances and/or norm equivalence				
Transcript	Original Quote	Translated Quote	Paraphrasing	Generalization
279, ll.23-25 Bosnian	„Also keine Krankheit, zu mindestens keine Bekannte“.	„Well, no disease, at least no known one”.	No known disease.	Absence of disease.
934, ll. 29-33 German	„Wird wahrscheinlich nicht sein, weil das nicht separabel ist. […]<br>Es ist (..) ne Krankheit, die wenn ich, wenn ich (…) Energie aufbringe, kann es so bleiben wie es ist. Und wenn ich schluderig werde, dann wird es schlechter, wird	“Will probably not be possible, because it is not separable. […] It is (..) a disease, of which I, when I (…) expend energy, it can be as it is. And if I get sloppy, then it gets worse, but it can never get better.”	Assessing higher SRH-status is not possible because of disease.	Absence of disease.

	aber nie besser.“			
398, l. 18 Turkish	„Sehr gut, weil keine Krankheit.“	“Very good, because no disease.”	I have no disease.	Absence of disease.

Table 3A Absence of subjective symptoms

Superordinate category<br><br>Absence of visible or non-visible disturbances and/or norm equivalence				
Transcript	Original Quote	Translated Quote	Paraphrasing	Generalization
279, l.23 Bosnian	„Es ist einfach so, dass ich keine Schmerzen habe“	„It is simply that I don’t have any pain”	Absence of pain	Absence of subjective symptoms
224 ll.19-20 German	„Ja, dass ich keine körperlichen Schmerzen habe“	„Yes, that I don’t have somatic pain”	Absence of somatic pain	Absence of subjective symptoms
827, l.21-22 German	„wie soll ich das sagen (…) weil ich keine Schmerze hab und weil ich (..) […] Keine Schmerzen habe“	„ how can I say (…) because I don’t have any pain and because I (..) […] Not having pain“	Absence of pain	Absence of subjective symptoms
130, l.6 Turkish	„Früher wo ich keine Zucker hatte war ganz anders, nicht wie jetzt. Schwitzt man nicht mehr“.	“Before, when I didn’t had sugar it was very different, not like now. You don’t sweat anymore”.	Absence of sweating as a symptom of a disease	Absence of subjective symptoms

Table 4A Presence of Subjective well-being

Superordinate category<br><br>Absence of visible or non-visible disturbances and/or norm equivalence				
Transcript	Original Quote	Translated Quote	Paraphrasing	Generalization
279, l.27 Bosnian	„Das der Kopf etwas ruhiger wird“	„That the head calms down more”	Absence of psychological distress	Subjective well-being

932, ll.39-40 German	„dass ich immer freudig durch die Welt lauf“	“that I always walk happily through the world”	Presence of a positive mood	Subjective well-being
102, ll-14-18 Bosnian	“ keine Sorgen, kein Kummer. (..) Nicht als Schmerzen aber zum allgemeinen Wohlbefinden.	“no worries, no sorrow. (..) Not as pain but for my overall well-being	Absence of negative feelings and moods and overall well-being	Subjective well-being
274, ll. 27-28 Turkish	„Aber es ist jetzt (..) es ist denn doch alles eher psychisch, die man, für die man was machen kann.“	“But it is now (..) It is all rather psychological, for that you can do something.”	Psychological wellbeing	Subjective well-being

Tables 5A-6A Freedom in the ability to act

Table 5A Performance level and role fulfilment

Superordinate category<br><br>Freedom in the ability to act				
Transcript	Original Quote	Translated Quote	Paraphrasing	Generalization
203, ll. 23-33 Turkish	„Auf meine tägliche, tägliche, Rhythmus. […] Ja, ob ich jetzt, meine tägliche Rhythmus so, wie gewöhnlich immer erlebe“	“On my daily, daily rhythm. […] Yes, whether I experience, my daily rhythm as I usually do.”	Being able to perform regular tasks in every-day life	Performance level and role-fulfilment
280, l. 15 Turkish	„Wenn ich arbeiten kann.“	“When I am able to work.”	Capability to work	Performance level and role-fulfilment
994, ll.29-30 German	„wenn ich gut angeben würde, würde ich vielleicht körperlich nicht diese Leistung bringen können.“	“If I would tick good, I maybe would not render this extent of physical fitness”	Physical fitness for very good	Performance level and role-fulfilment

827, l. 22<br>German	„ja und mobil bin“	“yes and being mobile”	Ability to walk	Performance level and role-fulfilment
152, l. 30<br>German	„Ich müsste, wollen wir mal so sagen, ich müsste wieder richtig gehen können.“	„I need to, let’s say it that way, I need to walk properly again.”	Ability to walk	Performance level and role-fulfilment

Table 6A No restrictions

Subordinate association dimension<br>Freedom in ability to act				
Transcript	Original Quote	Translated Quote	Paraphrasing	Generalization
203, ll. 23-28<br>Turkish	“einige Hindernisse, aufgrund meine, Beispiel meine Müdigkeit oder so gesundheitliche Problemen, genau an diesen Punkt gedacht, und wenn ich die denke, dann kann ich sagen, dass ich so eine gesunde Menschen bin.“	„certain restrictions, because of my, example my tiredness or health problems, I thought exactly of this aspect, and when I think this, then I can say, that I am a healthy person.”	No restriction due to disease or symptoms	No restrictions
430, ll.27-28<br>Bosnian	„ich kann gut essen und trinken (…)“	“[…] I can eat and drink well (…)”	No restriction in diet	No restrictions
492, ll.26-27<br>German	„Ich kann mich noch bewegen, ich kann noch Sport treiben, ich kann mich pflegen, und ich kann vieles mir noch ansehen.“	“I can still move, do sports, take care of me and see everything I want”	Not being restrict in every-day life	No restrictions

Tables 7A-9A Result of specific behaviours

Table 7A Healthy Lifestyle

Superordinate category<br>A result of specific behaviours				
Transcript	Original Quote	Translated Quote	Paraphrasing	Generalization
994, ll.21-22 German	„Was mir aufgefallen ist, dass ich doch sehr fit bin. […] dass ich […] körperlich fit zu sein.“	“What I realized is, that I am very fit. […] that I […] seem physically very fit.”	Because of my physical fitness.	Healthy lifestyle
494, ll.28-29 Turkish	„mehr Sport machen. Also mich mehr bewegen wahrscheinlich, ich glaube ja. Um noch fitter zu werden […].	“doing more sports. Well, doing more physical activities probably, I think yes. To become fitter.”	When I do more sports.	Healthy lifestyle
103, ll. 72-80 Bosnian	„So muss bisschen mehr auf Ernährung aufpassen […] Bewegung ja okay, bisschen mehr bewegen […] aber ein bisschen mehr Bewegung würde mir nicht schaden“	„I need to take care more of my diet […] physical activity yes, okay, a bit more physical activity […] but a bit more physical activity would not harm me.”	Improving healthy diet and sports	Healthy lifestyle

Table 8A Treatment

Superordinate category<br>A result of specific behaviours				
Transcript	Original Quote	Translated Quote	Paraphrasing	Generalization
784, l.36 Turkish	„Beispiel mein Rücken soll schön Massage gehen.“	„Example my back should go to a very nice massage.”	A massage against backpain.	Treatment

489, ll. 30-32 German	„dass man da vielleicht noch mal was unternimmt, dass ich Gymnastik kriege oder so etwas. Beweglicher werde […]“.	“maybe, taking action on this, such as getting gymnastic treatment or so. So that I get more agile […]”.	Gymnastics to increase mobility	Treatment
205, ll.27-29 Bosnian	„Ja, genau ich geh ja schon Kur, und Spritze und Massage [...]“	“Yes, exactly, I am already taking a cure, and injections and massage […]”	Cure and medication	Therapy
Treatment in form of religious or spiritual practices				
102, ll.151 153 Bosnian	„der Mensch besteht aus dem Körper und aus der Seele. Ist die Seele in Ordnung, ist der Körper auch in Ordnung. Und die Seele fütterst du mit anderen Dingen, also sprich mit Religion“	“the human consists of the body and the soul. If the soul is fine, then the body is fine as well. And you feed your soul with other things, such as religion”	You treat your body and soul with religious practices	Religious/Spiritual treatments
103, ll. 270-273 Bosnian	„Weil ich greife mehr so zu die andere Sachen wenn das so Beispiel wen Berkas Mutter mir was sagt, alte Sachen, alte Medizin und solche Sachen, weil diese Nebenwirkung heutzutage bei diese Tabletten und diese Pharmazie dann bin ich so ein bisschen skeptisch	“Because I take other things, that for example, if Berka’s mother tells me something, old things, old medicine and such things, because the side-effects nowadays with these pills and the pharmacy, then I am a little bit sceptical.”	Pharmaceutical medication is regarded with scepticism, while familiar, natural medication is favoured.	Traditional treatment

130, ll. 17-19 Turkish	„Es muss man immer aufpassen, immer weniger Essen und Sport viel laufen, ja (…) Beten“	“You always need to take care, always less eating and a lot of sports, walking a lot, yes (…) Praying”	You need to take care, do physical activity and pray.	Religious treatment

<u>Table 9A Relaxation</u>

Superordinate category<br><br><u>A result of specific behaviour</u>				
Transcript	Original Quote	Translation	Paraphrasing	Generalization
123, l.9-14<br><br>l.35-36 Bosnian	„Wenn ich (…) Freizeit genießen kann. […] Urlaub, ja. Wenn ich nach der Arbeit, wenn ich Urlaub habe, wenn ich mich erholt habe. Nach einer Woche, ein, zwei Wochen.<br>[…]<br>Ja. Weniger Stress. Weniger Arbeit. Acht Stunden dann zwischendurch auch mit […] unterwegs, das ist auch Stress“	“When I (…) can enjoy free-time. […] Holidays, yes. When I come home from work, when I have holidays, when I can recover myself. After one week, one, two weeks.<br>[…]<br>Yes, less stress. Less work. Eight hours of work and in between on the way with […], that is also stress”	Health is associated with free time and recovering from work.	Relaxation
333, ll. 31-36 Bosnian	Dann müsste man wirklich, ich sag mal so, auch im Urlaub sein oder wie auch immer.	Therefore, one needs to be, so to say, on holiday or whatever.	Being on holiday	Relaxation

224, l. 24 German	Weniger Stress zu haben.	Having less stress	Less stress	Relaxation
203, ll.36-38 Turkish	„Ausruhen, ausruhen, ausruhen. Einfach mehr Zeit für mich haben.“	“Relaxing, relaxing, relaxing. Simply having more time for myself.”	Relaxation	Relaxation

Table 10A Good relation to the social community

Superordinate category<br><u>Good relation to the social community</u>				
Transcript	Original Quote	Translated Quote	Paraphrasing	Generalization
479, ll. 35-40 Turkish	„Menschen Kontakt, soziale Verhalten ist wichtig nä? Das hab ich nicht immer, öfter. Soziales. Manchmal, ich hab Schwester hier, ich kann Sie nicht öfter besuchen, wegen Arbeit. Arbeit, nachhause und immer wieder nä. […] Das hängt alles zusammen. […] Sozialer Kontakt ist viel zu wenig.<br>I: Wenn das mehr wäre, würde es ihnen (…)<br>B: Ja würde das sehr gut gehen.“	“People contact, social behaviour is important, right? That is, what I don’t have very often. Social. Sometimes, I have sister here, I cannot visit her very often, because of work. Work, going home, and always, right? That is all linked together […] social contact is too little.”<br>I: If that would be more, than you would (…)<br>B: Yes, would be very good.”	Increasing social contact to family and friends.	Improving relation of social community.

666, ll. 19-24 Bosnian	“Also, wenn wir alle zusammen wären, wenn alles in der Familie nochmal alles (..) genau gesundheitlich fühle ich mich sowieso wohl, aber wenn wir zusammen wären öfter, nä. Das man sich eben sozusagen besser versteht und besucht und das, dass man mehr so miteinander sozusagen, mehr gemeinschaftlich so Sachen unternimmt und<br>I: Dann würde es Ihnen bessergehen als jetzt.<br>B: Ja arbeite so im Team so nä.“.	„Well, if we would all be together, if everything in the family, again everything (..) right, sanitarily I feel well either way, but if we would be together more often. That you, so to say, better understanding with each other and visiting each other and that, that you do, so to say, joint activities more often and<br>I: Then you would feel better than now?<br>B: Yes, working in a team. “.	Spending more time with the family and working together in a team	Improved relationship to family

B. <u>Supporting Quotes for lower SRH-options (good, fair, poor)</u>

Tables 11B-13B Visible or non-visible disturbances and/or deviation from the norm

<u>Table 11B Treatable or tolerable disease</u>

Superordinate category<br><u>Visible or non-visible disturbance and/or deviation from the norm</u>				
Transcript	Original Quote	Translated Quote	Paraphrasing	Generalization
224, ll.26-27 German	„(..) Erkältung. Eine Krankheit. Aber Erkältung, jetzt nicht eine langwierige, schwere Erkrankung,	“(..) Cold. A disease. But a cold, not a protracted illness, but a	A minor disease such as a cold.	Tolerable disease.

	sondern ne Erkältung oder Schnupfen oder so was“.	cold or sniffles or something like that.”.		
934, ll.24-26 German	„Ich habe Krankheiten, aber ich habe kein Krebs, zum Beispiel ich habe keine Krankheit, die töd(…), wo man sagt, die wird tödlich, also tödlich ausgehen. Ne. Also ich habe Krankheiten mit denen ich leben kann.“	“I have diseases, but I don’t have cancer, for example I don’t have diseases, which are leth(…), where you say, it will have an fatal, well a fatal ending. No. Well, I have diseases with which I can live.”	I don’t have any lethal disease but a disease with which I can live.	Tolerable disease.
494, ll.33-35 Turkish	“Wenn ich schon irgendwie regelmäßig, was heißt regelmäßig, schon in gewissen Abständen Schmerzen hätte, physische. Dann würde ich, also würde es mir schon gut gehen, aber eben nicht sehr gut.“	„If I would have somehow regularly, or what is regularly, at certain intervals pain, physical. Then I would, well I would still feel good, but not very good.”	If I would have physical pain in regular time intervals.	Tolerable disease.
279, ll. 31-33 Bosnian	“Ich glaube, wenn ich eine Krankheit bekomme, aber trotzdem eine Krankheit habe, wo es Medikamente oder Heilung oder keine Ahnung, oder nicht dauerhaft Schmerzen hab, dann bin ich immer noch wahrscheinlich, dann geht es mir immer noch gut.“	„I think, if I would get a disease, but still have a disease, for which medications or a cure or I don’t know, or not having constantly pain, then I am probably still, then I would still feel good.”	A disease that is treatable or tolerable and infrequent pain.	Tolerable or treatable disease.

102, ll. 40-46 Bosnian	“Also für mich persönlich, also ist Schnupfen oder Grippe oder so kein Hindernis arbeiten zu gehen. Das sind für mich Sachen gegenüber die man ankämpfen kann, einfach hart sein kann, also das ist nichts was einen in Bett zwingt, man muss nicht immer zum Arzt deswegen. Man fühlt sich zwar schlecht aber man macht trotzdem weiter, daher geht es mir immer sehr gut.“	„Well for me personally, well a cold or a flu or something like that, is not a hindrance to go to work. Those are things for me, which can be fought against, just can be tough, that is not something that forces you to bed, you don’t need to go to the doctor always, because of that. You might feel bad, but you continue, and that’s why I always feel very good.”	Minor symptoms can be fought against but there is no need to always visit the doctor, even when I feel bad I need to continue, that’s why I always feel good	Tolerable symptoms or disease

<u>Table 12B Presence of disease</u>

Superordinate category<br><u>Visible or non-visible disturbance and/or deviation from the norm</u>				
Transcript	Original Quote	Translated Quote	Paraphrasing	Generalization
123, l. 38 Bosnian	„Krankheit oder sowas“	“disease or something like that”	Presence of disease	Presence of disease
934, ll.35-36 German	„Joa (…) das wär bei der nächsten Untersuchung, das der Arzt sagt, jetzt ist es schlechter geworden.“	“Well (…) this would be for the next medical examination, that the doctor says, that it got worse”	Medical check-up proves a deterioration of the disease	Presence of disease

182, ll. 35-36 German	„wenn da jetzt noch ein paar Sachen zukommen, noch weitere Krankheiten […]“	“if further diseases come along”	Further diagnosis of diseases	Presence of disease
274, ll. 42-44 Turkish	„Ja naja, wenn ich jetzt so weiter mache, dann würde es sich verschlechtern. […] Aber wenn ich jetzt so weitermachen, was mein Problem betrifft, wird es weniger gut, wenn nicht schlecht.“	“Yes, well. If I would continue as now, then it will get worse. […] But if I would continue like that, concerning my problem, it will get less good, if not even bad.”	Continuing presence and deterioration of disease	Presence of disease

Table 13B Subjective symptoms

Superordinate category<br>Visible or non-visible disturbance and/or deviation from the norm				
Transcript	Original Quote	Translated Quote	Paraphrasing	Generalization
913, l. 23 Bosnian	„Also, wenn ich, also die Schmerzen größer werden.“	„Well, if I, well if the pain gets worse. “	Increasing symptoms	Subjective symptoms
205, ll.24-25 Bosnian	„Rücken, wenn ich habe Verletzung in meinem Rücken“	„Back, when I have injury in my back”	Back pain	Subjective symptoms
274, ll. 27-28 Turkish	„aber körperlich habe ich auch paar kleine Probleme.	„but physically, I also have a few small problems.“	Somatic malfunction	Subjective symptoms
222, l.33 German	„Ja, dann müssten sich diese	„Yes, then these symptoms need to intensify.“	Increasing symptoms	Subjective symptoms

	Beschwerden verstärken.“			
152, ll.26-28 German	„Weil ich im Augenblick sehr viel Probleme habe mit der Luft und dadurch, dass ich (....) dass ich viel wegen Sauerstoff im Blut habe, habe ich Probleme beim Gehen, meine Beine sind schwer.“	„Because currently I have a lot of problems with the air and because I have (…) I have a lot of oxygen in my blood, I have problems with walking, my legs are heavy.”	Somatic symptoms	Subjective symptoms

Tables 14B-15B Limitation in the ability to act

Table 14B Hindrance in performance level and role-fulfilment

Superordinate category<br><br>Limitation in the ability to act				
Transcript	Original Quote	Translated Quote	Paraphrasing	Generalization
444, ll.35-36 Turkish	„Also, dass man überhaupt nicht mehr aufstehen will, dass man überhaupt nicht mehr zur Arbeit gehen will, dass man irgendwie kein bock hat.	“Well that you don’t want to wake up anymore, that you don’t want to work, that you somehow don’t feel like it.”	Not being able to wake up and go to work to fulfil social roles	Hindrance in performance level and role-fulfilment
994, ll.29-30 German	„Wenn ich gut angeben würde, würde ich vielleicht körperlich nicht diese Leistung bringen können.“	“If I would tick good, I would not render this extent of physical fitness.”	Not being able to perform physical fitness	Hindrance in performance level and role-fulfilment
203, ll.41-44 Turkish	“Ich glaube, wenn ich nicht so viel, nicht	“I believe, if I would not have that much, no	Not being able to render energy to do something	Hindrance in performance

	mehr Energie habe, dann (…)“	energy anymore, then (…)”		level and role fulfilment
333, ll.38-45 Bosnian	“Ja also, passieren gut ist, wenn man wirklich nicht krank ist, man geht doch arbeiten“	“Well, yes happens good is, when you are really not ill, you go to work”	Illness is defined as not being capable to work	Hindrance in performance level and role fulfilment

Table 15B Dependency

Superordinate category<br>Limitation in the ability to act				
Transcript	Original Quote	Translated Quote	Paraphrasing	Generalization
205, ll. 36-37 Bosnian	Das ich dann ständig Schmerzmittel nehmen muss, jetzt ist noch ruhig.	That I constantly need to take painkillers, now it is seemingly quit	Increasing intake of medication.	Frequent intake of medication.
489, ll. 40-41 German	auf fremde Hilfe angewiesen bin.	Being dependent on external help	Dependency on others.	Dependency on others.

Tables 16B-17B A result of specific behaviours

Table 16B Unhealthy lifestyle

Superordinate category<br>A result of specific behaviours				
Transcript	Original Quote	Translated Quote	Paraphrasing	Generalization
103, ll. 96-97 Bosnian	„Wenn ich mich weniger bewege, mehr essen würde ich mich weniger gut fühlen.“	“When I decrease my physical activity, if I would eat more, I would feel less good”	Less exercise and more eating.	Unhealthy lifestyle

101, ll.21-22 Turkish	„wenn ich mich weniger bewegen würde und noch mehr zunehmen würde, dann wäre das Katastrophal.“	“if I would decrease my physical activity and would increase in weight, that would be disastrous.”	Less physical fitness and increase in weight.	Unhealthy lifestyle
130, l. 21 Turkish	“Wenn ich viel Zucker esse oder Fett und (mhm) zunehmen“	„When I eat a lot of sugar or fat and (mhm) gain weight”	Unhealthy diet and weight increase	Unhealthy lifestyle

Table 17B Work

Superordinate category<br>A result of specific behaviours				
Transcript	Original Quote	Translated Quote	Paraphrasing	Generalization
784, ll.27-45 Turkish	„weil mein Rücken schmerzen. […] weil ich arbeite, halb fünf aufstehen […] Deswegen bin ich, mein Rücken total. […] Ja, viel arbeiten, dann […] acht Stunden, dann neun Stunden arbeiten ist sehr viel.“	“because my back is paining […] because I am working, waking up half past four […] That’s I am, my back is totally […] a lot of working then […] eight hours, then nine hours working is a lot.”	More work lead to backpain.	Working

Table 18B Disturbed well-being of the social community

Superordinate category<br>Disturbed well-being of the social community				
Transcript	Original Quote	Translated Quote	Paraphrasing	Generalization
430, ll.43-46 Bosnian	„Naja, wenn man Probleme hat, so (…) Familie so, was weiß ich. Wenn in Bosnien Familie schlecht geht, dann geht mir natürlich auch nicht so. finanziell auch, helfen dies und das und so“	„Well, if you have problems, like (…) family, whatever. When in Bosnia family is unwell, then I as well of course don’t feel good. Also, financially, helping here and there and so on. “	Threats in well-being or financial problems in my family in Bosnia.	Disturbed well-being of the social community
280, ll. 23-24 Turkish	„Auch wieder mein Umfeld, besonders Familie nicht gut geht, dann ja, ich bin dann halt auch nicht so gut“	“Again, my surrounding, especially my family not well. Then yes, then I am also not good”	Disturbed well-being of the surrounding especially the family.	Disturbed well-being of the social community
