# Supplementary material for: Exploring ethnic differences in understanding of self-rated health among persons of Turkish, Bosnian and German origin
Source: BMC Res Notes. 2018 Dec 18;11:903. doi: 10.1186/s13104-018-4019-9 (PMC6299561; doi:10.1186/s13104-018-4019-9)
Supplement: Supplementary file 2 — Additional file 2. Allocation of subordinate definition categories among the higher (excellent, very good, good) and lower SRH options (good, fair, poor) for each ethnic group. [file 13104_2018_4019_MOESM2_ESM.pdf]

Additional File 2 - Allocation of subordinate definition categories among higher and lower SRH options for each ethnic group

A. Allocation of subordinate definition categories among excellent, very good and good SRH for each ethnic group

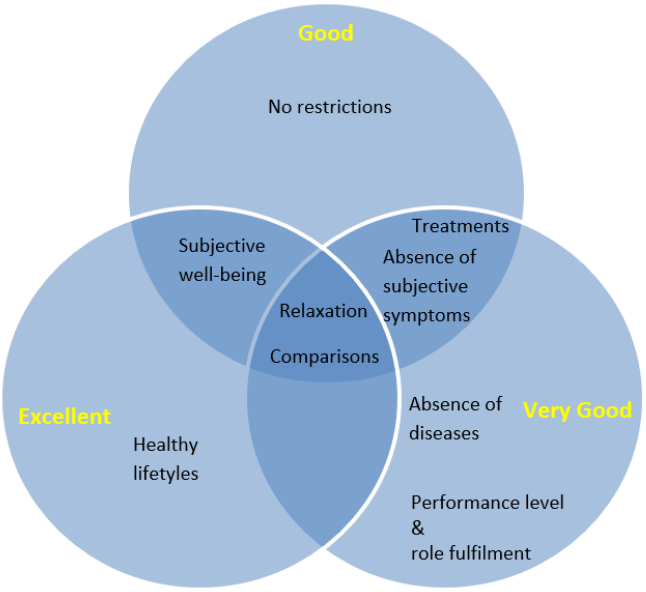

Figure 1A Definitions for excellent, very good and good health among the German study group

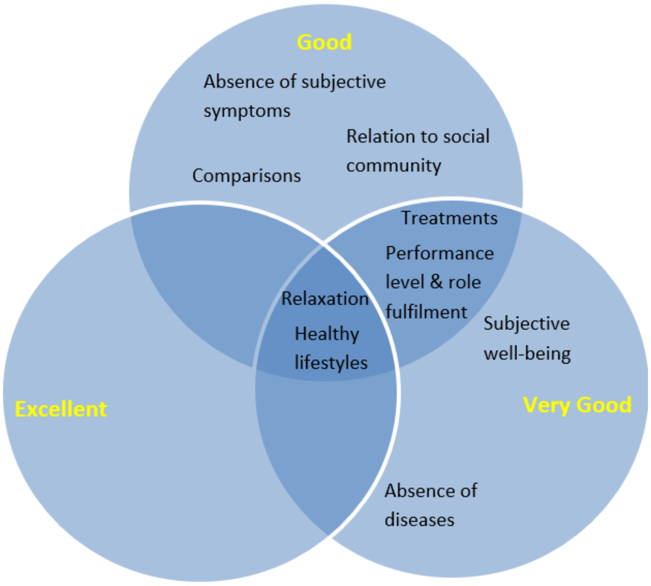

Figure 2A Definitions for excellent, very good and good health among the Turkish study group

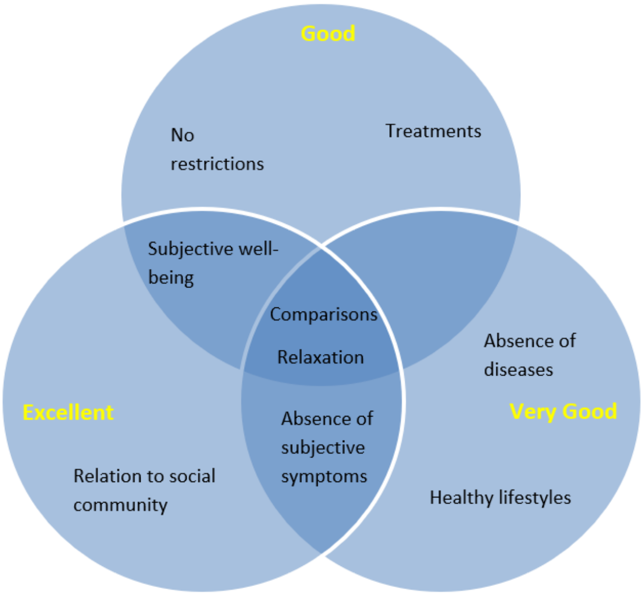

Figure 3A Definitions for excellent, very good and good health among the Bosnian study group

B. Allocation of subordinate definition categories among good, fair and poor SRH for each ethnic group

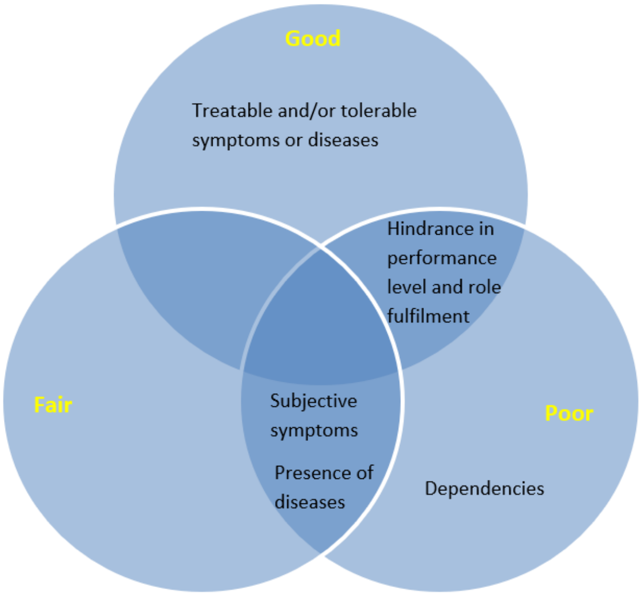

Figure 1B Definitions for good, fair and poor health status among the German study group

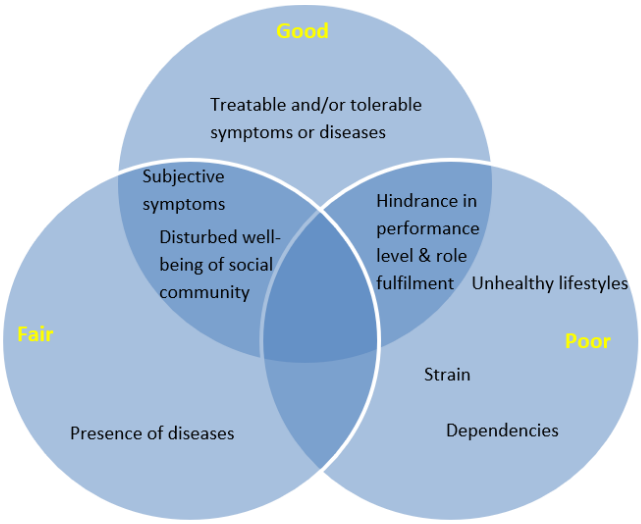

Figure 2B Definitions for good, fair and poor health status among the Turkish study group

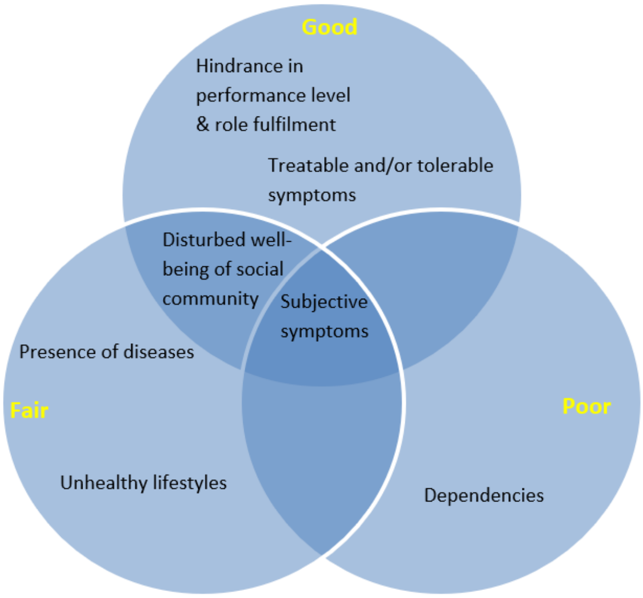

Figure 3B Definitions for good, fair and poor health status among the Bosnian study group
